# Passive Wireless Partial Discharge Sensors with Multiple Resonances

**DOI:** 10.3390/mi15050656

**Published:** 2024-05-17

**Authors:** Zhenheng Xu, Bing Tian, Shiqi Guo, Qingan Huang, Lifeng Wang, Lei Dong

**Affiliations:** 1 Key Laboratory of MEMS of the Ministry of Education, School of Electronic Science & Engineering, Southeast University, Nanjing 210096, China; xuzh4@csg.cn (Z.X.); guoshiqi_1003@163.com (S.G.); hqa@seu.edu.cn (Q.H.); wanglifeng@seu.edu.cn (L.W.); 2 CSG Sensing Technology (Guangdong) Co., Ltd., Shenzhen 518067, China; tianbing1227@163.com

**Keywords:** multi-resonant, inductor–capacitor sensor, partial discharge detection, wireless sensing

## Abstract

Partial discharge (PD) is the dominant insulating defect in Gas-Insulated Switchgear (GIS). The existing detection methods are mainly divided into built-in wire-connected disk antennas with destructive drilling and external ultra-high frequency antennas with poor anti-interference ability. This research introduces a passive wireless PD sensor implanted inside GIS on the observation window. The sensor is implemented by a sheeting branch-inductor with multiple resonances which is able to enhance detection sensitivity. A coaxially aligned readout circuit, positioned outside the GIS, interrogates the PD sensor to wirelessly obtain the PD signal. The proposed sensing scheme improves signal-to-noise ratio and ensures minimal disruption to the electric field distribution inside GIS. An experimental setup was established in a controlled laboratory environment to benchmark the multi-resonant sensor against the commercial UHF sensor. A 2.5-times enhancement of signal strength was observed. Since our sensor was implanted inside the GIS, a high signal-to-noise ratio (68.82 dB) was obtained. Moreover, we constructed a wireless calibration test to investigate the accuracy of the proposed sensor. The precision of the signal test was as high as 0.72 pC. The pulse phase distribution information was collected to demonstrate a phase-resolved partial discharge (PRPD) pattern. The experiment results validate the effectiveness of the proposed method and demonstrate excellent performance in PD detection.

## 1. Introduction

Gas-insulated switchgear (GIS), characterized by its excellent sealing capabilities, robust anti-interference properties, and notably low failure rates, has gained widespread adoption in power systems, representing a crucial component of the electrical infrastructure [1]. However, the intricate nature of GIS equipment poses a significant challenge in faulty localization and maintenance, markedly more complex than that associated with conventional electrical apparatus. Hence, the prompt detection of faults within GIS systems is of paramount importance [2].

Partial discharge (PD) serves as a key early indicator of insulation degradation in GIS. The occurrence of PD within GIS is typically accompanied by a range of physical manifestations, including luminescence, thermal effects, mechanical vibrations, and electromagnetic emissions [3,4]. These phenomena provide a foundation for various PD detection methodologies.

Techniques such as infrared thermography allow for the identification of heat signatures associated with PD. However, this optical detection method is unable to detect partial discharge when the source of the discharge is outside the detection field of view [5]. However, the ultrasonic method still has its drawbacks, such as reliance on the path between the PD source and the sensor, high attenuation, and lower sensitivity for PD detection and localization in the field [6]. The pulse current method requires an ideal testing environment and is susceptible to interference from the external electromagnetic environment. It cannot achieve localization of partial discharges. Meanwhile, the chemical detection method is more commonly used for offline testing, requiring the extraction of the internal gas from the GIS equipment for laboratory analysis of its composition [4]. The ultrasonic method is operationally flexible, as it can be installed both inside and outside the GIS equipment without being affected by electromagnetic noise in the field environment [7,8].

Additionally, UHF detection techniques are employed to recognize the electromagnetic signals emanating from PD, facilitating comprehensive and effective monitoring of GIS equipment health [9]. Currently, the UHF detection method is extensively utilized for the reception of electromagnetic wave signals emanating from PD, enabling the live detection and real-time surveillance of these discharges within GIS. This method can be categorized into two types based on sensor installation locations: external and internal [10].

External UHF sensors offer several advantages, such as versatility in installation, non-intrusive nature that preserves the internal electric field of GIS, and no operational impact on the equipment. These characteristics make them well suited for online monitoring applications [11,12]. However, existing external sensors typically encounter challenges like bulkier structural dimensions, vulnerability to external noise, and reduced sensitivity [13]. Conversely, internal sensors are distinguished by their high sensitivity and minimal susceptibility to environmental interferences [14,15]. The design of internal sensors, however, involves complex considerations. Incorporating a balun is necessary for achieving balanced feeding and impedance matching, which consequently elevates the profile of the built-in antenna. This increase in size can adversely affect the GIS’s internal electric field distribution. Additionally, the wired connection of the internal antenna raises concerns regarding potential sulfur hexafluoride gas leaks [13].

A notable issue with the UHF detection method is the broad frequency spectrum of partial discharge electromagnetic signals. Most of the energy in these signals is concentrated in the lower frequency range. However, UHF antennas are typically operational only within the 300–1500 MHz frequency band [16]. This limitation means that the strength of PD signals within this specific frequency band is relatively weak [17], leading to an underutilization of the full spectrum of the PD electromagnetic waves. Hence, antennas with frequency within 100 MHz are proposed for detecting PD signal [18].

To effectively address the demands for online monitoring of PD in GIS within practical field applications, it is imperative to develop a sensor that is both compact and structurally straightforward, yet highly sensitive and non-destructive. This sensor should be capable of detecting low-frequency PD signals wirelessly.

Addressing these challenges, this study innovatively integrates LC passive wireless sensors with PD detection techniques. LC passive wireless sensors offer several key advantages: diminutive size, uncomplicated design, theoretically unlimited lifespan, and suitability for enclosed environments, aligning well with the necessities of GIS PD detection [19,20,21].

**Figure 1:**
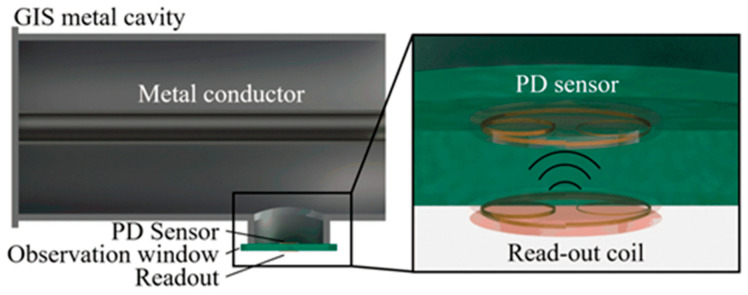
Installation of multi-resonant PD sensors and read-out coil on GIS. The passive wireless PD sensor is implanted inside the GIS to achieve highly accurate detection with high SNR. A readout circuit is utilized to wirelessly interrogate the PD sensor via the observation window.

This paper delves into the application of LC passive wireless sensor technology, particularly tailored to the unique attributes of PD electromagnetic wave signals. We have developed an LC passive wireless PD sensor that is installed inside the GIS observation window. This sensor is characterized by multiple resonance peaks within the 0–800 MHz frequency range. A coaxially aligned readout coil is externally mounted on the GIS, as depicted in Figure 1. Comprehensive simulation analysis was conducted, followed by the construction of a laboratory test system to evaluate its performance. This system’s detection capabilities were then benchmarked against commercial UHF sensors.

The study successfully demonstrates the feasibility of passive wireless measurement of PD in GIS, fulfilling the criteria of miniaturization, high precision, simplistic design, and preservation of the GIS’s internal electric field integrity. This advancement represents a significant stride in GIS partial discharge sensor technology.

## 2. Sensor Design and Fabrication

### 2.1. PD Spectrum Analysis

This investigation undertakes a comprehensive examination of electromagnetic wave signals generated during PD within GIS. The cardinal insulation anomalies intrinsic to GIS encompass four principal categories: (1) Fixed Metal Protrusion Defects; (2) Free Metal Particle Defects; (3) Loose Connections between Metal Components and the Ground; and (4) Air Gap Defects between the Insulator and the High-voltage Conductor. These identified insulation defects precipitate four discrete types of PD: tip discharge, particle discharge, surface discharge, and air gap discharge.

**Figure 2:**
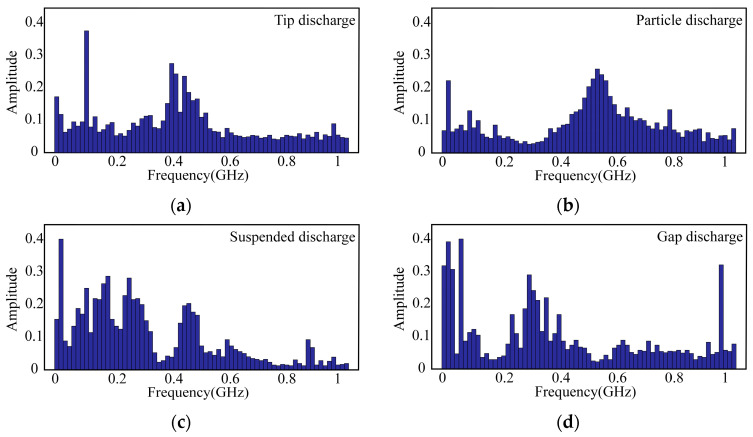
The frequency spectrum of four types of partial discharge. (**a**) Tip discharge. (**b**) Particle discharge. (**c**) Suspended discharge. (**d**) Gap discharge.

The ensuing segment of this scholarly exposition conducts a meticulous spectral analysis of signals corresponding to these four discharge typologies, as delineated in Figure 2 [22]. The graphical portrayal reveals conspicuous dissimilarities in the waveform of electromagnetic wave signals instigated by distinct insulation anomalies. Specifically, the energy spectrum of electromagnetic wave signals generated by tip discharge predominantly localizes within the frequency band of 300–600 MHz; particle discharge evinces signal energy concentration primarily within the range of 400–800 MHz; surface discharge demonstrates signal energy concentration in the frequency bands of 100–300 MHz and 400–500 MHz; and air gap discharge elicits signal energy primarily within the frequency ranges of 0–50 MHz and 200–400 MHz.

### 2.2. Design and Fabrication of Multi-Resonant PD Sensor

In light of the frequency characteristics exhibited by electromagnetic wave signals emanating from the aforementioned four categories of PD in GIS, their signal energy predominantly aligns within the 0–800 MHz frequency spectrum, conveniently falling within the operational frequency range of LC passive wireless sensors [23]. This scholarly contribution integrates LC sensors with the realm of PD, introducing an innovative multi-resonant PD sensor founded on LC passive wireless sensors.

**Figure 3:**
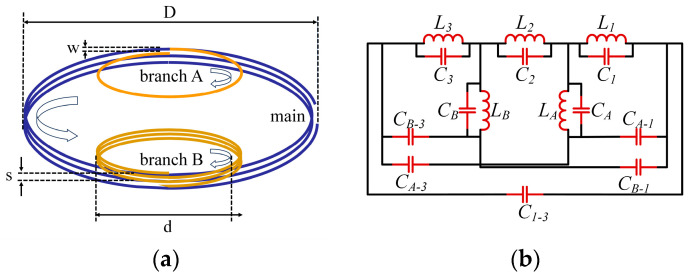
Schematic diagram of multi-resonant PD sensor. (**a**) Structural overview of the sensor. (**b**) Schematic of multi-resonant PD sensor.

The sensor configuration adopts a branch inductor structure, illustrated in Figure 3, wherein one end of the branch interfaces with the main inductor while the other remains suspended. This arrangement engenders a closed resonant circuit through capacitive coupling and inductive mutual inductance, with each branch assumed as a pivotal role in fostering multiple resonances. The unfolded architecture of the sensor, as depicted in Figure 3a, encompasses a principal branch and two ancillary branches labeled A and B. These components are intricately wound in opposite directions to configure spiral inductors. The primary inductor adopts a counterclockwise spiral orientation, while the two branch inductors exhibit a clockwise spiral direction. Adhering to this prescribed configuration, additional branches can be incorporated to augment the repertoire of resonant frequencies. This innovative design not only capitalizes on the inherent frequency characteristics of PD in GIS, but also facilitates the potential expansion of resonant frequencies through judicious structural modifications.

The sensor orchestrates the creation of multiple resonant circuits by integrating inductor coils, parasitic capacitors, and capacitive coupling between inductor coils, as visually represented in Figure 3b. This intricate configuration culminates in the attainment of diverse resonant frequencies. The delineation in Figure 3a elucidates that branches *A* and *B* effectively partition the main inductor into three distinct sections, corresponding to the equivalent circuit expounded in Figure 3b. This circuit comprises primary inductances ($L_{1}$, $L_{2},\;$and $L_{3}$) and branch inductances ($L_{A}$ and $L_{B}$), along with their respective parasitic capacitors ($C_{1}$, $C_{2}$, $C_{3}$, $C_{A},$ and $C_{B}$). Additionally, capacitive couplings ($C_{A-1}$, $C_{B-1}$, $C_{A-3}$, $C_{B-3}$ and $C_{1-3}$) interconnect these components, collectively shaping the fundamental resonant circuits. This intricate interplay of inductor coils and capacitors within the sensor structure not only ensures the establishment of multiple resonant frequencies, but also underlines the meticulous arrangement and connectivity of the sensor components.

**Figure 4:**
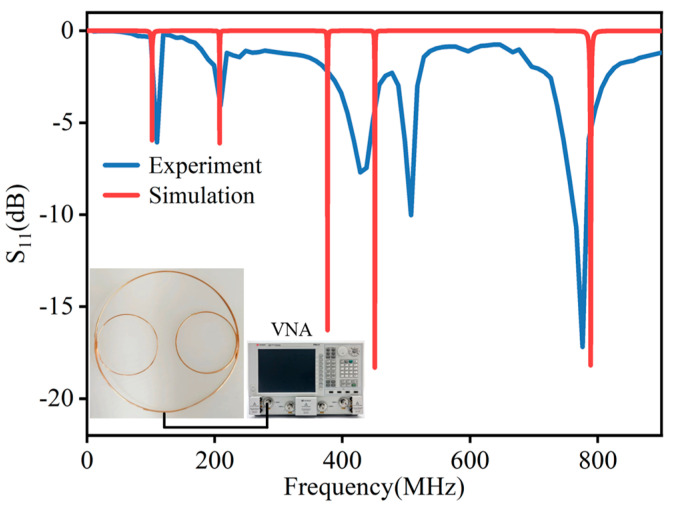
S11 parameter of multi−resonant PD sensor.

In order to achieve enhanced precision in detecting PD while concurrently minimizing the sensor’s volume to prevent any disruption in the electric field distribution within GIS, the investigated sensor has been meticulously wound with a copper wire with a diameter of 0.5 mm. Table 1 offers the dimensional specifications of the designed multi-resonant PD sensor. The physical configuration of the sensor is visually represented in Figure 4. Notably, the thickness of the proposed sensor is as thin as 2.12 mm, a crucial feature ensuring that its installation within GIS has no adverse impact on the electric field distribution.

We constructed a simulation in ADS software 14.1 to investigate the spectrum performance of the sensor. The values of the parameters in the schematic setup are listed in Table 2. The corresponding parameters in simulations are decided by the theoretical calculation of the coils, such as the partial inductance and parasitic capacitance. The results of the S11 parameter in Figure 4 indicate five discernible resonance peaks within the 0–800 MHz range, precisely situated at 101.90 MHz, 207.60 MHz, 376.70 MHz, 450.80 MHz, and 788.90 MHz. We employed a vector network analyzer (VNA) to measure the resonant frequency as well. The testing result is illustrated in Figure 4. The measured S11 parameters also exhibit five resonant peaks in the 0–800 MHz spectrum, located at 109.5 MHz, 209 MHz, 427.9 MHz, 507.5 MHz, and 776.2 MHz, respectively, with the highest value recorded at −17.18 dB. The five resonant peaks displayed by the S11 parameter match the five main resonant circuits shown in the equivalent circuit diagram.

There is discernible deviation between the simulation and measurement results, accompanied by slight disparities in the positions of the resonance peaks. The utilization of electromagnetic simulation software cannot cover all the parasitic components in practical detection. Moreover, manufacturing discrepancies, such as imprecise soldering connections between branch inductors and main inductors, lead to the mirror. Additionally, measurement errors, encompassing calibration inaccuracies and cable losses, can further impact the overall precision of the results. Therefore, a comprehensive understanding of these factors is imperative for interpreting and contextualizing the observed differences between simulated and measured results.

### 2.3. Internal Electric Field Distribution in GIS

**Figure 5:**
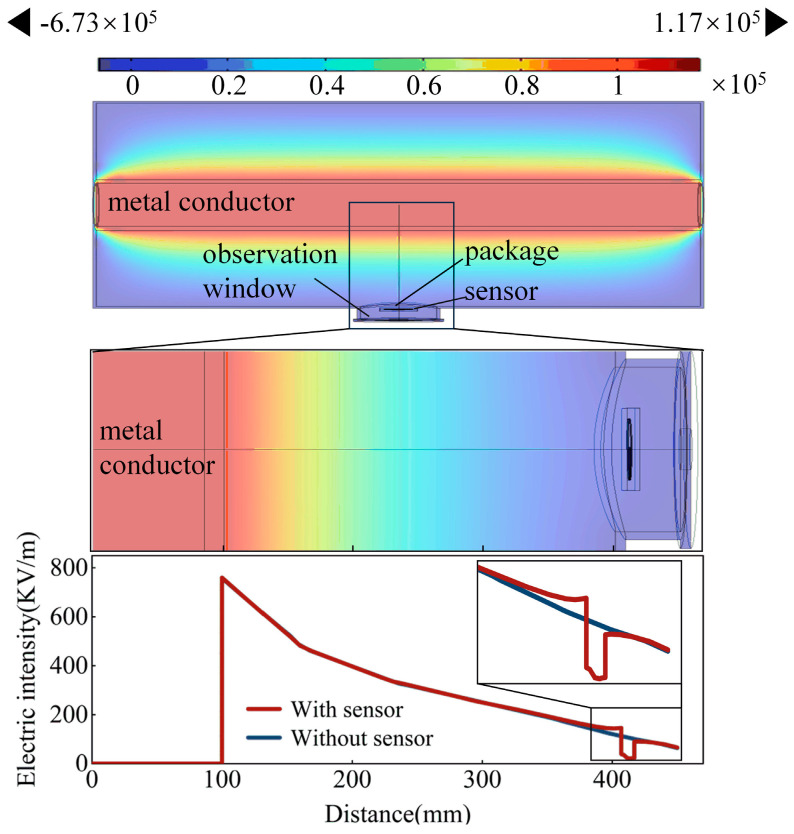
The influence of sensors on the internal electric field distribution in GIS.

In this investigation, simulations were executed by using the COMSOL software 6.2. The simulation model, depicted in Figure 5, employed a single-phase coaxial configuration for the GIS. The conductor featured an outer diameter of 200 mm and a thickness of 8 mm, while the GIS cavity possessed an outer diameter of 816 mm with an 8 mm thickness. A voltage of 110 kV was applied to the conductor during the simulation. Specifically, for the current simulation, only a segment of the GIS cavity (1.6 m in length) was taken into consideration. The structural parameters of the sensors remained consistent with the detailed specifications outlined in the preceding section. The dielectric constant of the copper conductor was set to 1 × 10^9^.

The sensor was strategically positioned at the observation window of the GIS and insulated with epoxy resin. The package thickness was 5 mm. The results in Figure 5 illustrate the distribution of electric potential and field strength.

An examination of Figure 5 discloses that in the absence of sensors, the electric field strength exhibits a gradual decline along the radius of the tank. The continuous electric field is punctuated by the observation window because of the dielectric medium at that specific location. Upon the introduction of sensors, three discernible discontinuities in electric field strength appear, which are the breakpoints occurring at the sensor encapsulation, the sensor itself, and the observation window.

In addition, the electric field strength at the sensor location remains lower than that of the situation without sensors. This observation substantiates the assertion that the installation of sensors exerts no discernible impact on the internal electric field distribution of the GIS and metal conductor and does not introduce interference with its normal operational behavior. The findings underscore the efficacy of the sensor placement in detecting PD without adversely affecting the overall electric field dynamics within the GIS.

## 3. Experiments and Results

### 3.1. Wireless Experiment Setup

We utilize a PD simulation source, capable of precisely controlling the discharge amount, to ascertain the wireless passive PD sensor’s performance. The challenge lies in accurately controlling the discharge amount when inducing PD through the application of high voltage to real insulation defects. To overcome this limitation, the present study draws upon Conference International des Grands Reseaux Electriques(CIGRE)‘s recommendation of employing a UHF detection system for on-site verification, based on equivalent discharge electric field strength [1]. A pulse PD source, offering precise control over the discharge amount, serves as the signal source. The RF coaxial line is employed to convey the pulse current signal to the transmitting antenna, thereby converting the pulse signal into an electromagnetic wave signal.

**Figure 6:**
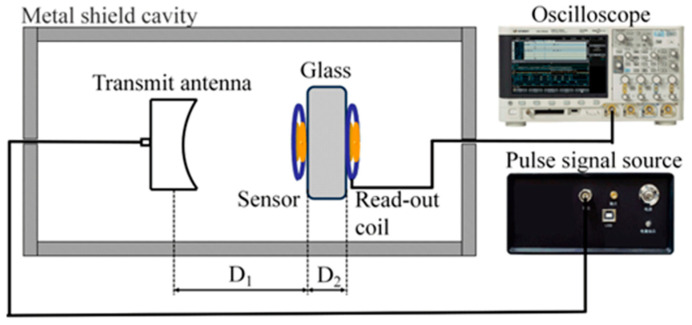
The partial discharge test system is designed with D_1_ = 5 cm and D_2_ = 3 cm.

We employed a metal tank with an outer wall diameter of 360 mm, a wall thickness of 30 mm, and a height of 500 mm as the electromagnetic shielding cavity. Inside the shield cavity, the transmitting antenna, multi-resonant PD sensor, and readout coil are coaxially aligned. The RF coaxial line transfers the collected signal to the oscilloscope. Simultaneously, a 10 cm × 10 cm × 3 cm glass is employed to mimic a GIS observation window inserted between the wireless PD sensor and readout coil, as shown in Figure 6.

### 3.2. Results of the Multi-Resonance PD Sensor

**Figure 7:**
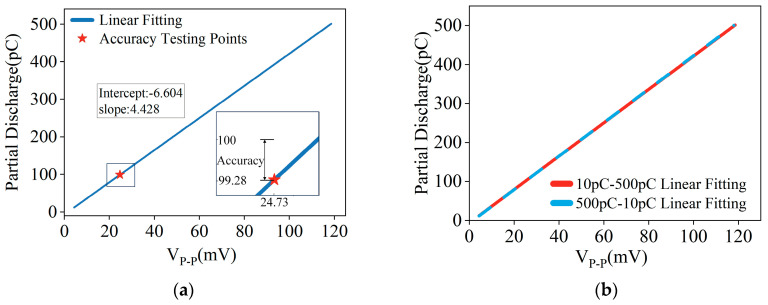
Fitting function between peak-to-peak value of multi-resonant PD sensor signal and discharge amount: (**a**) accuracy at discharge amount of 100 pC; (**b**) the linear fitting of the increase and decrease in the amount of discharge.

We conducted a precision test on the proposed PD sensor. In this study, accuracy was conceptualized as the magnitude of deviation between the actual value and the measured value, essentially denoting the absolute error. This definition frames the methodology for the precision assessment of the multi-resonance PD sensor. The calibration and precision testing process encompasses the following stages:

(1) Calibration: the initial step involves calibrating the sensor using charge quantities to generate pulse signals with discharge powers of 10 pC, 20 pC, 50 pC, 150 pC, 200 pC, 300 pC, 400 pC and 500 pC, constituting eight distinct test points. At each test point, the voltage peak-to-peak *V_PP_* at the read-out coil are recorded using an oscilloscope. For each test point, ten sets of data are collected, and their mean value, denoted as *U*, is calculated. These average amplitude values, in conjunction with the corresponding discharge quantities *Q*, are then used to establish a fitting function. This function calibrates the relationship between the voltage peak-to-peak *V_PP_* and the discharge quantity *Q* for the multi-resonance PD sensor, facilitating the determination of the discharge quantity from the sensor’s voltage peak-to-peak *V_PP_*.

(2) Measurement: following calibration, the sensor’s measurement accuracy is evaluated. The pulse signal source is adjusted to a discharge power of 100 pC. Utilizing the proposed PD sensor, this discharge power is detected, and the peak signal values are recorded. At this test point, ten data sets are again recorded to derive an average. The measured discharge energy value, *Q*’, is obtained through the pre-established fitting function. The accuracy of the measurement is then ascertained by calculating the difference between this value and the standard 100 pC. Both the fitting function and the results of the measurement test are illustrated in the accompanying Figure 7.

When the pulse signal source output a discharge power of 100 pC, the sensor recorded an average peak-to-peak voltage *V_PP_* = 24.73 mV. Utilizing the established fitting function, this voltage signal corresponds to a calculated discharge power of 99.28 pC. Crucially, the disparity between this computed value and the actual discharge power of 100 pC was 0.72 pC. In actual measurement, no hysteresis effect was found. The linear fitting curve from 500 pC down to 10 pC is basically consistent with the fitting curve from 10 pC up to 500 pC.

### 3.3. Comparison with UHF Sensor

To substantiate the enhanced signal strength in the low-frequency band of PD electromagnetic wave spectrum by the proposed PD sensor, a comparative analysis was conducted. The study involved the test results of both the multi-resonant PD sensor and the commercial UHF sensor (antenna type: butterfly, gain: +5 dBi). The calibration of sensor detection ability was based on the peak-to-peak value of the voltage signal observed on oscilloscope. A higher peak-to-peak voltage V_PP_ indicates a more excellent detection capability.

**Figure 8:**
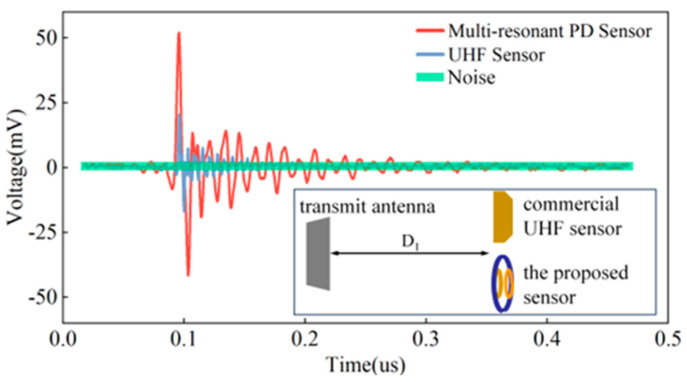
Comparison of PD signals detected by multi−resonance PD and UHF sensor. Inset shows the experiment setup with D_1_ = 5 cm.

Figure 8 presents received signal strength comparisons between our PD sensor and the commercial UHF sensor. The experimental findings reveal that, under identical distances and discharge power settings, the signal received by the proposed sensor (V_PP_ = 93.67 mV) is almost 2.5 times of the voltage received by the commercial UHF sensor (V_PP_ = 37.33 mV). The signal-to-noise ratio SNR of the signal received by the proposed sensor is 68.82 dB, while SNR of the commercial UHF sensor is 50.42 dB. This observation indicates that the detection capability of the multi-resonance PD sensor is more potent compared to that of the commercial UHF sensor.

### 3.4. Generation of PRPD Pattern

**Figure 9:**
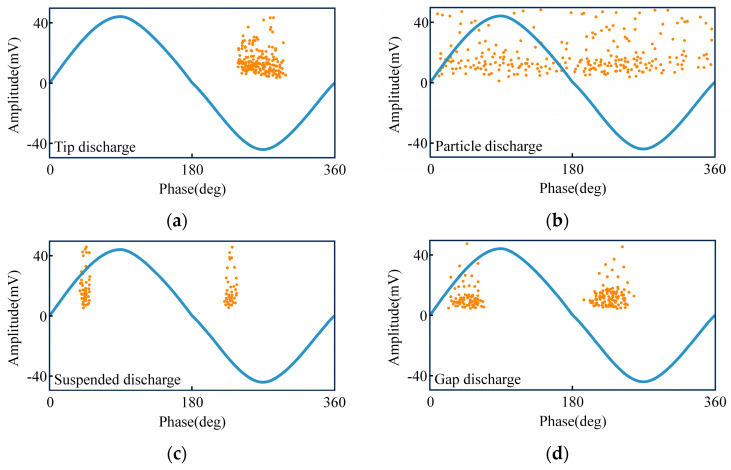
PRPD of four types of partial discharge. (**a**) Tip discharge. (**b**) Particle discharge. (**c**) Suspended discharge. (**d**) Gap discharge.

The phase-resolved partial discharge (PRPD) pattern is a pivotal tool for classifying PD phenomena. Distinct PD types manifest in varied phases of the power frequency sine wave. This study explores the efficacy of multi-resonant PD sensors in accurately detecting and categorizing PD types by generating representative PRPD spectra. The experimental setup includes four PD sources: tip, particle, suspension, and gap discharges, each emitting pulse signals congruent with their characteristic discharge patterns. For each type, 50 power frequency cycles worth of discharge signals were collected to construct the PRPD plot, as depicted in the corresponding Figure 9.

Figure 9a illustrates that tip discharge pulses predominantly occur in the 225°–315° phase range. Conversely, as Figure 9b demonstrates, particle discharge pulses lack a discernible pattern, appearing randomly throughout the power frequency cycle. Figure 9c indicates that suspension discharge pulses are limited to the 45° and 315° phases. Finally, Figure 9d shows that gap discharge pulses are primarily concentrated in the 30°–60° and 210°–240° phase intervals.

The PRPD spectra, derived from signals captured by the multi-resonant PD sensor system, accurately mirror the pulse phase distribution characteristics inherent to each discharge type. This performance is comparable to that of other sensors. Consequently, it can be inferred that multi-resonant PD sensors are viable for the identification and categorization of PD patterns.

## 4. Conclusions and Discussion

This paper presents an innovative integration of LC passive wireless sensors with PD detection, introducing a technology that utilizes multi-resonant LC sensors specifically designed for this purpose. The technology successfully achieves multiple resonant frequencies spanning the 0–800 MHz range, utilizing a dual-branch inductor structure. This range effectively encompasses the frequency band where PD signals exhibit a pronounced energy distribution, thus facilitating high-precision detection of such discharges.

A key area of ongoing research is whether the incorporation of additional branches leading to a greater number of resonant frequencies could further enhance the capability to detect PD. From the perspective of magnetic coupling energy transfer, the wireless signal receiver should be matched with the sensor probe to achieve the maximum energy transfer requirement [24]. When the readout coil and the sensor match, the energy transfer efficiency is highest. Here in our work, our readout coil adopts the same structure as the sensor to improve impedance matching. Concurrently, this study conducted simulations of the internal electric field within GIS, affirming that the proposed sensor’s lightweight design does not adversely impact the electric field distribution in GIS.

An experimental system for PD detection was established, which validated the sensor’s robust detection capability. Notably, the sensor demonstrated an accuracy of 0.72 pC, along with an ability to precisely preserve the phase distribution information of PD pulses. This feature is pivotal for the subsequent identification of different types of PD in experimental analyses. In summary, this study not only advances PD detection technology with its innovative multi-resonant PD sensor design but also establishes a significant benchmark in precision and effectiveness for future research and applications in this field.

## Figures and Tables

**Table 1:** Dimensions values of multi-resonant PD sensor.

Symbol	Parameter	Value
D	Diameter of main inductor	75 mm
d	Diameter of main inductor	32 mm
N	Turns of main inductor	3
n_A_	Turns of brunch inductor A	1
n_B_	Turns of brunch inductor B	3
w	Diameter of copper wire	0.5 mm
s	Spacing of copper wire	0.06 mm

**Table 2:** The schematic parameters of multi-resonant PD sensor in simulations.

Parameter	Value	Parameter	Value	Parameter	Value
$L_{1}$	0.117 μH	$C_{1}$	1 pF	$C_{A-1}$	0.1 pF
$L_{2}$	0.234 μH	$C_{2}$	1.1 pF	$C_{B-1}$	0.1 pF
$L_{3}$	1.056 μH	$C_{3}$	0.1 pF	$C_{A-3}$	0.1 pF
$L_{A}$	0.067 μH	$C_{A}$	0.5 pF	$C_{B-3}$	0.1 pF
$L_{B}$	0.565 μH	$C_{B}$	1 pF	$C_{1-3}$	1 pF

## Data Availability

The original contributions presented in the study are included in the article, further inquiries can be directed to the corresponding author.
